# Retracted: Resveratrol Ameliorates Motor Neuron Degeneration and Improves Survival in SOD1^G93A^ Mouse Model of Amyotrophic Lateral Sclerosis

**DOI:** 10.1155/2019/4729651

**Published:** 2019-10-29

**Authors:** BioMed Research International

BioMed Research International has retracted the article titled “Resveratrol Ameliorates Motor Neuron Degeneration and Improves Survival in SOD1^G93A^ Mouse Model of Amyotrophic Lateral Sclerosis” [1]. Based on concerns raised on PubPeer, the article was found to contain image duplication and splicing as follows:

- Part of the second panel in the second row (SDH) of Figure 3 is the same as the first one, but rotated and flipped.
- The second panel (*β*-actin) in Figure 4(a) is the same as the second panel (*β*-actin) in Figure 5(f).
- There is a splicing in the first two panels in the last row (COX) of Figure 3, where the second panel completes the first one, if placed below it.

Additionally, image duplication was found between this article [1] and another article by the same group of authors [2] as follows:

- The first panel in Figure 2(b) in [1] is the same as panel (d) in Figure 3(a) in [2].
- Part of the third panel in the first row of Figure 3 (HE) in [1] is the same as that of Figure 4 in [2], but flipped vertically.
- Part of the second panel in the second row (SDH) of Figure 3 in [1] is the same as the first panel in the last row of Figure 4 in [2], but flipped horizontally.

We asked the authors for their explanation, and they stated the first author carried out the experiments of both articles [1, 2] at the same time and cut the PVDF membrane into three pieces and applied exposure, respectively, for the Sirt1, beta-actin, and PARP proteins. They added that the experiment of [1] was completed in a hurry, and that the mistakes occurred in this process lead to the image duplication. The authors apologized and requested retraction.
